# Mitochondrial miRNA miR-134-5p Play Oncogenic Role in Clear Cell Renal Cell Carcinoma

**DOI:** 10.3390/biom15030445

**Published:** 2025-03-20

**Authors:** Tao Shen, Wei Wang, Haiyang Wang, Xinyi Zhu, Guoping Zhu

**Affiliations:** 1 Anhui Provincial Key Laboratory of Molecular Enzymology and Mechanism of Major Metabolic Diseases, College of Life Sciences, Anhui Normal University, Wuhu 241000, China; wang_hy@ahnu.edu.cn (H.W.); zhuxinyi@ahnu.edu.com (X.Z.); 2 Anhui Provincial Engineering Research Centre for Molecular Detection and Diagnostics, College of Life Sciences, Anhui Normal University, Wuhu 241000, China; 3 Department of Geriatrics, Gerontology Institute of Anhui Province, Centre for Leading Medicine and Advanced Technologies of IHM, The First Affiliated Hospital, Division of Life Sciences and Medicine, University of Science and Technology of China, Hefei 230026, China; ww571@mail.ustc.edu.cn

**Keywords:** mitochondrial miRNA, miR-134-5p, clear cell renal cell carcinoma, cancer, cell migration

## Abstract

Mitochondrial miRNAs (mitomiRs), which are miRNAs that located within mitochondria, have emerged as crucial regulators in a variety of human diseases, including multiple types of cancers. However, the specific role of mitomiRs in clear cell renal cell carcinoma (ccRCC) remains elusive. In this study, we employed a combination of experimental and bioinformatic approaches to uncover the diverse and abundant subcellular distribution of miRNAs within mitochondria in ccRCC. Notably, RNA sequencing after mitochondrial fractionation identified *miR-134-5p* as a miRNA predominantly detected in the mitochondria of 786O cells, and its expression is significantly upregulated compared to that in 293T cells. Differential expression and survival analyses from TCGA reveal that the upregulation of *miR-134-5p* is prevalent and closely associated with poor survival outcomes in ccRCC patients. Functionally, exogenous overexpression of *miR-134-5p* mimics promotes migration in both 786O and Caki-1 cells. Mechanistically, overexpressing the *miR-134-5p* mimic dramatically downregulates the mRNA levels of *CHST6*, *SFXN2*, and *GRIK3*, whereas the *miR-134-5p* inhibitor markedly upregulates their expression. Notably, these target mRNAs also predominantly detected in the mitochondria of 786O cells. The downregulated expression signatures of *CHST6*, *SFXN2*, and *GRIK3* are also closely correlated with poor survival outcomes in ccRCC patients. Taken together, our work identifies a novel mitomiR, *miR-134-5p*, in ccRCC, provides potential targets that could serve as effective biomarkers for ccRCC diagnosis and prognosis, and opens new avenues for understanding the mitomiR-directed regulatory network in ccRCC progression.

## 1. Introduction

Clear cell renal cell carcinoma (ccRCC) is the most prevalent subtype of kidney cancer, accounting for approximately 70–80% of all renal malignancies [1]. This cancer is characterized by distinct metabolic alterations, including the dysregulation of the tricarboxylic acid cycle and increased reliance on aerobic glycolysis, which contribute to tumorigenesis and progression [2]. Despite advances in surgical techniques and systemic therapies, the prognosis for patients with advanced ccRCC remains poor, with a 5-year survival rate of only 12–30% [3]. The pathogenesis of ccRCC is complex, involving genetic alterations such as mutations in the VHL gene, which play a critical role in the regulation of hypoxia-inducible factors and subsequent metabolic pathways [4]. Although recent research has highlighted the urgent need for novel biomarkers that can aid in early diagnosis, prognostication, and personalized treatment strategies, current biomarkers, such as serum creatinine and lactate dehydrogenase, are insufficient for fully capturing the metabolic heterogeneity of ccRCC [5,6]. Therefore, the identification of new, reliable biomarkers is crucial for improving clinical outcomes and facilitating targeted therapies.

Mitochondria, as central hubs of cellular metabolism and energy production, play a pivotal role in the metabolic reprogramming observed in ccRCC [2,7,8]. Given the profound metabolic dysregulation in ccRCC, understanding the molecular mechanisms governing mitochondrial function is essential for uncovering novel therapeutic targets and biomarkers. Mitochondrial microRNAs (mitomiRs), a distinct class of small non-coding RNAs located within the mitochondria, have emerged as key regulators of mitochondrial function and cellular metabolism [9]. Unlike conventional miRNAs, which primarily function in the cytoplasm, mitochondrial miRNAs are involved in modulating mitochondrial functions directly and influencing cellular processes from within the organelle [10]. In the context of cancer, several specific mitochondrial miRNAs, such as miR-1, miR-181c, and miR-495, have been implicated in the regulation of metabolic reprogramming and mitochondrial dynamics. For instance, miR-1 has been shown to influence oxidative phosphorylation and apoptosis in various cancer types, while miR-181c is involved in regulating mitochondrial biogenesis and energy metabolism [11,12]. Furthermore, miR-495 has been linked to the modulation of glycolytic pathways, highlighting the intricate relationship between mitochondrial miRNAs and cancer metabolism [13]. Despite the growing recognition of mitomiRs as critical regulators of mitochondrial function and metabolism in cancer, their specific roles in ccRCC remain largely unexplored. Therefore, further investigation into the roles of mitochondrial miRNAs in ccRCC is essential for determining their potential as therapeutic targets and biomarkers for this aggressive cancer type.

In this study, we uncovered a diverse and abundant subcellular distribution of miRNAs within the mitochondria of ccRCC cells. Notably, we identify *miR-134-5p* as a mitomiR that is predominantly expressed in the mitochondria of 786O cells, with significantly higher levels compared to 293T cells. Our differential expression and survival analyses reveal that the upregulation of *miR-134-5p* is prevalent in ccRCC and closely associated with poor survival outcomes in patients. Through the exogenous overexpression of *miR-134-5p* mimics, we observed its roles in promoting cellular migration in both 786O and Caki-1 cell lines. Additionally, we identified a *miR-134-5p*-directed regulatory network, comprised with *CHST6*, *SFXN2*, and *GRIK3* in ccRCC progression. Collectively, our findings identify *miR-134-5p* as a novel mitomiR in ccRCC, providing potential targets that could serve as effective biomarkers for ccRCC diagnosis and prognosis. This work opens new avenues for understanding the mitomiR-directed regulatory network in ccRCC progression, highlighting the importance of mitochondrial miRNAs in the pathophysiology of this aggressive cancer type.

## 2. Materials and Methods

### 2.1. Cell Culture

The HEK293T (hereafter referred as 293T), 786O, and Caki-1 cells were obtained from Procell (Wuhan, China). 293T Cells are derived from human embryonic kidney (HEK) cells, which were highly transfectable due to the expression of SV40 large T antigens, making them suitable for viral production and transient gene expression studies [14]. The 293T cells were cultured in Dulbecco’s modified Eagle’s medium (DMEM) (vivacell, Shanghai, China), containing 10% fetal bovine serum (vivacell, Shanghai, China) and 1% penicillin-streptomycin (WISENT, Nanjing, China). Caki-1 cells were derived from human skin metastasis of renal cell carcinoma, which are used as a model for studying metastatic renal cell carcinoma and exhibit epithelial morphology [15]. The Caki-1 cells were cultured in RPMI-1640 medium (Procell, Wuhan, China), containing 10% fetal bovine serum (FBS, Gibco, Waltham, MA, USA) and 1% penicillin-streptomycin. The 786O cells were derived from human renal cell carcinoma, which were widely used as a model for studying renal cell carcinoma and exhibit typical characteristics of clear cell renal carcinoma [15]. The 786O cells were cultured in RPMI-1640 medium (Procell, Wuhan, China), containing 10% fetal bovine serum (FBS, Gibco, USA) and 1% penicillin-streptomycin.

### 2.2. Mitochondrial Isolation and RNA Sequencing

Mitochondria were isolated using Cell Mitochondria Isolation kit (Beyotime Biotechnology, Shanghai, China) according to the manual. Briefly, the procedure involved first collecting 293T and 786O cells. After centrifuging, the supernatant was discarded, and the cells were resuspended in 1 mL of mitochondrial isolation reagent, and incubated on ice for 15 min. Then, cell suspension was transferred to a glass homogenizer and homogenized for 30 strokes. The number of homogenization strokes is optimized based on trypan blue staining of cells. Homogenization is continued until >50% of cells are stained positive, avoiding over-homogenization which damages mitochondria. Next, the homogenate is centrifuged at 1000× *g* for 10 min at 4 °C. The supernatant is carefully transferred to another tube and centrifuged at 3500× *g* for 10 min at 4 °C. The precipitate was the isolated mitochondria. The supernatant was collected and centrifuged to obtain cytosolic control components.

Then, the RNA was extracted from samples after mitochondrial isolation with TRIzol reagent (Ambion, Austin, TX, USA). The quality and quantity of the RNA were assessed using a One Drop^®^ OD-1000 Spectrophotometer (Nanjing Wuyi Corporation, Nanjing, China) and an Agilent 2100 Bioanalyzer (Agilent Technologies, Santa Clara, CA, USA). The RNA molecules in a size range of 18–30 nt were enriched by polyacrylamide gel electrophoresis (PAGE). Then, the 3′ adapters were added and the 36–44 nt RNAs were enriched. The 5′ adapters were then ligated to the RNAs as well. The ligation products were reverse transcribed by PCR amplification and the 140–160 bp size PCR products were enriched to generate a cDNA library and sequenced using Illumina NovaSeq X Plus by Gene Denovo Biotechnology Co. (Guangzhou, China).

### 2.3. Public Data Download and Preprocessing

The RNA-sequence data, clinical information, and phenotype information of ccRCC patients were downloaded from the TCGA (https://portal.gdc.cancer.gov/, accessed on 15 May 2023). Before analyzing the dataset, patients with missing pathological diagnostic data and corresponding clinical information were excluded. Overall, the ccRCC cohort downloaded from the TCGA database for this study included 615 samples and their corresponding phenotypic data, including 71 normal counts and 544 tumor counts.

### 2.4. Identification of Differentially Expressed mRNAs and miRNAs

To identify differentially expressed mRNAs and miRNAs, we utilized the “DESeq2” R package to analyze RNA-seq and miRNA-seq data. Differential expression analysis was performed between tumor and normal tissues in ccRCC, as well as between 786O and 293T cells at the cellular level. For both analyses, miRNAs and mRNAs were considered differentially expressed if they met the following criteria: |log2(fold change)| > 1 and *p* value < 0.05. These thresholds were chosen based on widely accepted standards in transcriptomic and survival analysis studies [16,17,18,19], as well as a balance between biological relevance and statistical rigor.

To further refine our analysis, we integrated miRNA-seq data and clinical information from TCGA-retrieved ccRCC patients and normal controls (accessible at https://portal.gdc.cancer.gov/, accessed on 15 May 2023). We focused on differentially expressed mitomiRs derived from the following two distinct cohorts: “786O versus 293T” at the cellular level and “Tumor versus Normal” at the tissue level. This dual-level approach allowed us to isolate mitomiRs with consistent dysregulation across both cellular and tissue contexts.

To identify mitomiRs with prognostic value, we performed Cox regression analyses based on the expression levels of miRNAs. miRNAs were considered significant if they were associated with overall survival (OS) in TCGA-retrieved ccRCC patients (*p* < 0.05). These miRNAs were classified as either protective factors (hazard ratio, HR < 1) or adverse factors (hazard ratio, HR > 1). By intersecting the prognostic miRNAs identified through Cox regression with the differentially expressed mitomiRs, we further screened out prognostic dysregulated mitomiRs.

### 2.5. Identification of Prognosis-Related Genes

Univariate Cox regression analysis was used to identify prognosis-related genes. Prognosis-related genes were analyzed using the R package “UpSetR” with *p* < 0.05 indicating statistical significance.

### 2.6. Functional Enrichment Analysis

Aiming to ascertain which biological processes and functions in which miR-134-5p-targeted genes were mainly enriched, we performed gene ontology (GO) analysis using the R package “clusterprofiler” [20]. *p* value < 0.05 was regarded as statistically significant.

### 2.7. RNA Isolation and Reverse Transcription (RT)—Quantitative PCR (qPCR)

RNA was extracted from cell samples using Trizol Reagent (Ambion, USA) according to the manufacturer’s instructions. One Drop^®^ OD-1000 Spectrophotometer (Nanjing Wuyi Corporation, China) was used to measure RNA concentration and purity. The RNA was reverse-transcribed using HiScript II One Step RT-PCR Kit (Vazymen, Nanjing, China) and the Bulge-Loop miRNA qRT-PCR primer set for miR-134-5p and U6 (RiBoBio, Guangzhou, China) according to the manufacturer’s instructions. The following qPCR analysis was performed using SYBR^®^ Green Master Mix (Vazyme, China) on Light Cycle^®^ 96 (Roche, USA). The primers for the real-time PCR are listed in Table 1. Bulge-Loop^TM^ miRNA qRT-PCR Starter Kit (RiBoBio, China) was used to detect miR-134-5p. Fold changes were determined using the relative quantification 2^−ΔΔCT^ method.

### 2.8. Western Blot Assay

The 3× Sample Buffer (1 M Tris-HCl pH of 7.4, 6% SDS, 0.03% bromophenol blue, 34.5% glycerol) was diluted to the 1.5× sample buffer. Cells were lysed in 1.5× sample buffer and boiled for 10 min at 100 °C. After quantification, whole-cell lysates or cell fractions were separated by SDS-PAGE under denaturing conditions and transferred to PVDF membranes (Millipore, USA). The membranes were blocked in 5% BSA (Sangon, Shanghai, China) for 1 h at room temperature, and then incubated at 4 °C overnight with primary antibody β-Tubulin (Proteintech, Chicago, IL, USA), Fibrillarin (Proteintech, Chicago, IL, USA), CS (Proteintech, Chicago, IL, USA), and Cytochrome C (Proteintech, Chicago, IL, USA). The PVDF membranes were then incubated with secondary antibodies conjugated with horseradish peroxidase (Proteintech, USA). Immunoreactive proteins were visualized using the SuperSignal^®^ West Femto Maximum Sensitivity Substrate (Thermo Scientific, Waltham, MA, USA) on Tanon 5200Muti chemiluminescence gel imaging system (Tanon, Shanghai, China).

### 2.9. miRNA Mimic Overexpression

For cell transfection experiment, miRNA mimics manufactured by RiboBio (Guangzhou, China) were transfected into cells using Hieff Trans in vitro siRNA/miRNA transfection reagent (YEASEN, Shanghai, China), according to the manufacturer’s instructions.

### 2.10. Wound Scratch Assay

Cells were plated into 6-well plates and incubated at 37 °C in 5% CO_2_ until reaching 100% confluence and straight scratches were made. After, we washed it with 1× PBS, new medium was added, and the cells were further cultured for 24 h. Each experiment was performed in triplicate wells to ensure reproducibility. The cells from three views in each well were photographed at 0 h and 24 h. The migration area was assessed using Image-pro plus 6.0. The average area of the three counted views per well was calculated, and the data from triplicate wells were pooled for statistical analysis. *p* < 0.05 considered statistically significant.

### 2.11. Transwell Assay

Transwell chambers, inserting with an 8 μm pore size in 24-well cell culture plates (Corning, New York, NY, USA), were used. Each experiment was performed in triplicate chambers to ensure reproducibility. A total of 2 × 10^4^ of cells were suspended in 200 μL serum-free media and added to the upper chamber. Complete medium containing 20% fetal bovine serum (1 mL) was added to the bottom chamber as a chemo-attractant. The chambers were incubated in cell culture incubator for 24 h. After incubation, the non-migrated cells in the upper chamber were removed with cotton swabs. The membranes were fixed with ethanol absolute and stained with 0.25% crystal violet. The migrated cells were counted in three randomly selected fields per chamber under a light microscope, and the average number of cells per field was calculated. Data from triplicate chambers were pooled for statistical analysis. *p* < 0.05 considered statistically significant.

### 2.12. Statistical Analysis

Statistical analysis was carried out using Microsoft Excel 2021 software, GraphPad Prism 9.0.2, and R software 4.3.1. The analysis of variance (ANOVA) method was employed to statistically analyses multi-group data, while the Wilcoxon rank sum test was used to compare two groups. Statistically significant differences were marked as *: *p* < 0.05, **: *p* < 0.01, and ***: *p* < 0.001.

## 3. Results

### 3.1. Expression Profiles of Mitochondrial miRNAs in 293T and 786O Cells

**Figure 1:**
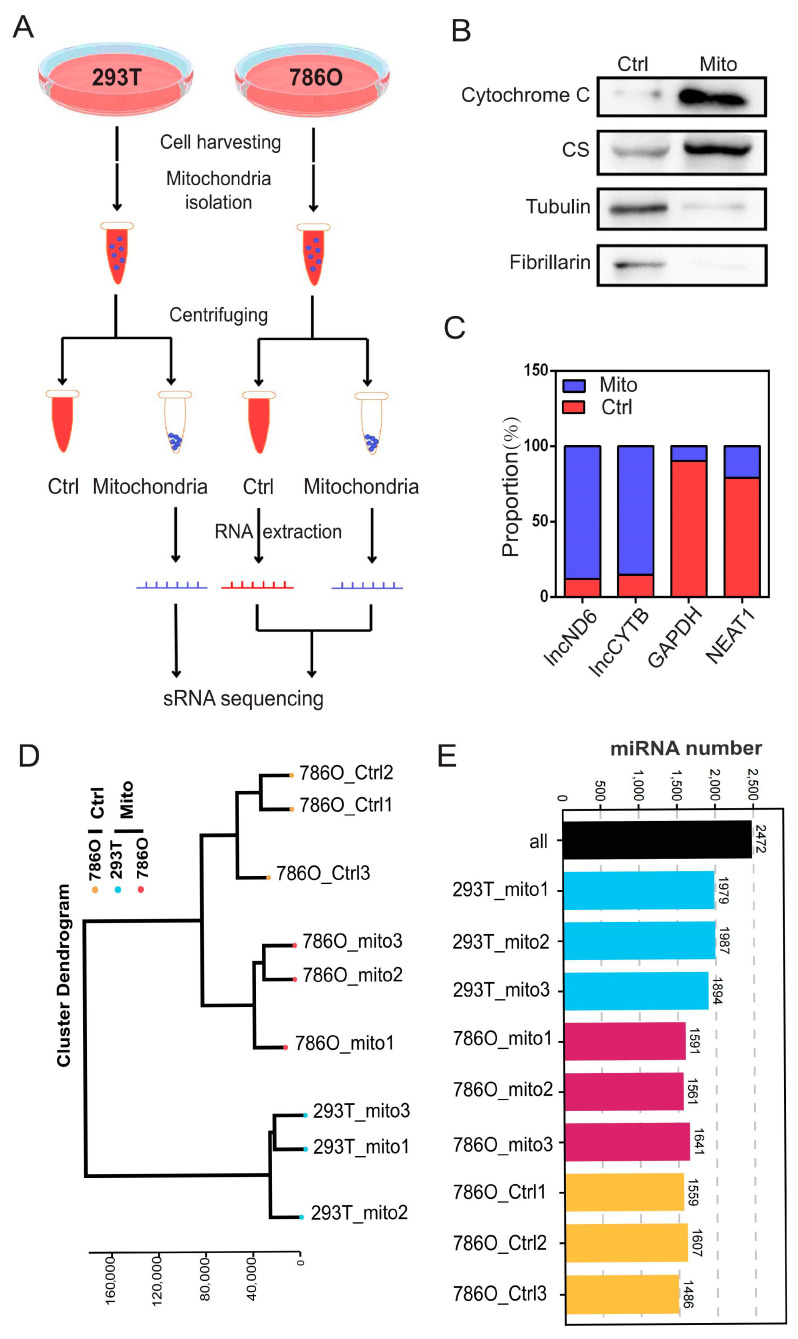
Expression landscape of mitochondrial miRNAs in 293T and 786O cells. (**A**) A schematic view of experimental outline for identifying the mitochondrial miRNAs in the indicated 293T and 786O cell. (**B**) The subcellular distributions of protein cytochrome C, CS, tubulin, and fibrillarin determined by fractionationing assay in 786O cell (n = 3). (**C**) The subcellular distributions of RNA *lncND6*, *lncCYTB*, *GAPDH*, and *NEAT1* determined by fractionationing assay in 786O cell (n = 3). (**D**) The cluster dendrogram of the indicated samples. (**E**) Statistical chart of miRNA identification results. Original Western blot images can be found in Supplementary File S1.

To systematically investigate the role of mitomiRs in ccRCC, we isolated mitochondria and the corresponding control components from ccRCC cell line 786O and a control human renal epithelial cell line 293T, as illustrated in Figure 1A. The purity of the isolated organelle components was further validated using their specific protein and RNA markers: mitochondria (proteins: cytochrome C, citrate synthase (CS); RNAs: *lncND6*, *lncCYTB*), cytosol (protein: tubulin; RNA: *GAPDH*), and nuclei (protein: fibrillarin; RNA: *NEAT1*) (Figure 1B,C and Figure S1A,B). Following this, we conducted small RNA sequencing on nine samples from the following three distinct groups: the mitochondrial component of 293T cells, the control component of 786O cells, and the mitochondrial component of 786O cells. Quality control analyses, encompassing individual expression distribution assessments, technical replicate evaluations, and clustering analyses, were applied to validate these sequencing results (Figure 1D and Figure S2A–J). These analyses indicated the consistency and reproducibility of the replicates within each group. Through comprehensive small RNA sequencing, we identified a total of 2472 organelle-associated microRNAs. Among them, an average of 1953 mitomiRs were detected in 293T cells, while an average of 1570 mitomiRs were identified in 786O cells (Figure 1E).

### 3.2. miR-134-5p Stands Out for Its Unique Upregulated and Oncogenic Characteristics in ccRCC

**Figure 2:**
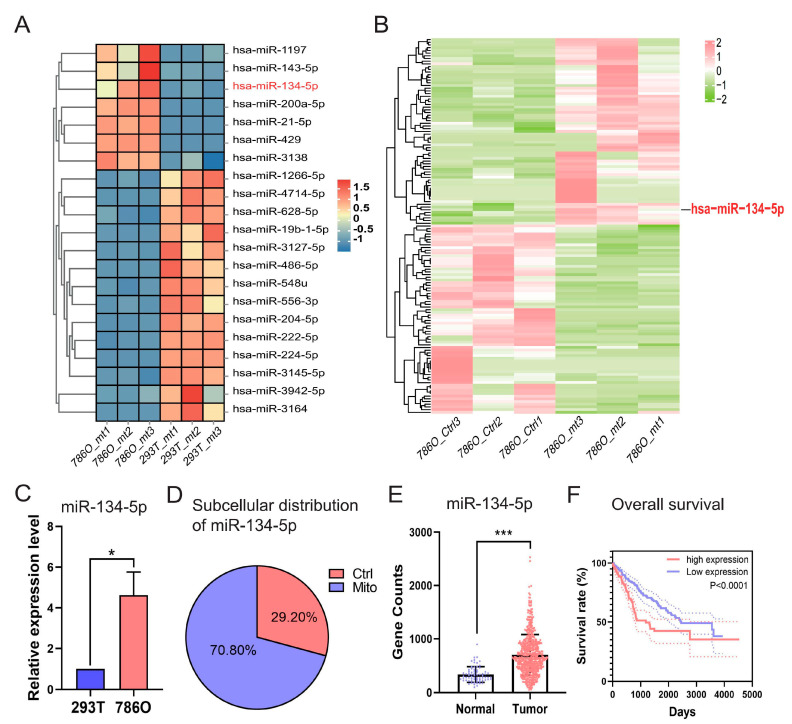
Identification of *miRNA-134-5p* as a prognostic dysregulated mitomiR dominantly distributed in the mitochondria of 786O cells. (**A**) Heatmap shows the expression of prognostic mitomiRs in the indicated 293T and 786O cells. (**B**) Heatmap shows the distribution of mitomiRs in the indicated 786O cellular components. (**C**) Relative expression of *miR-134-5p* in the indicated 293T and 786O cells (n = 3). (**D**) The subcellular distributions of *miR-134-5p* determined by fractionationing assay in 786O cell (n = 3). (**E**) The expression of *miR-134-5p* in TCGA-retrieved normal and tumor ccRCC samples (n (normal) = 71, n (tumor) = 544). (**F**) Survival analysis of miR-134-5p low- and high-expression subgroups in the TCGA-retrieved ccRCC patients (n (low) = 253, median survival time = 2454 days; n (high) = 253, median survival time = 1191 days; HR = 1.963 (1.413–2.727)). Data are shown as means ± SEM. *** *p* < 0.001, and * *p* < 0.05.

As illustrated in Figure S3A, we conducted a comprehensive comparative analysis by integrating sRNA-seq data from 786O and 293T cells, and miRNA-seq data and clinical information obtained from TCGA-retrieved ccRCC patients and normal controls (accessible at https://portal.gdc.cancer.gov/, accessed on 15 May 2023). Our focus was on differentially expressed mitomiRs derived from the two distinct cohorts of “786O versus 293T” at the cellular level (Figure S3B and Table S1) and “Tumor versus Normal” at the tissue level (Figure S3C and Table S2). This approach allowed us to isolate differentially expressed mitomiRs at both the cellular and tissue levels. To further identify those with prognostic value, we performed Cox regression analyses based on the expression levels of miRNAs, ultimately determining 46 significant miRNAs that influenced the overall survival (OS) of TCGA-retrieved ccRCC patients. These miRNAs acted either as protective factors (hazard ratio, HR < 1) or as adverse factors (hazard ratio, HR > 1) (Figure S3D and Table S3). By intersecting the indicated 46 prognostic miRNAs with the aforementioned differentially expressed mitomiRs, 21 dysregulated mitomiRs were further screened out and designated as prognostic dysregulated mitomiRs (pdmt-miRNAs) (Figure 2A and Table S4).

Among these pdmt-miRNAs, we observed that *miR-134-5p* was uniquely upregulated in the mitochondria of 786O cells when compared to either the mitochondria of 293T cells or the control components within 786O cell (Figure 2A,B and Tables S4 and S5), indicating its potential roles in regulating ccRCC progression. To further validate its dysregulation and subcellular distribution, we performed RT-qPCR after fractionation assays in both 293T and 786O cells. Our results confirmed that *miR-134-5p* was significantly upregulated in the mitochondria of 786O cells, regardless of whether it was compared to 293T cells or the control components within 786O cells (Figure 2C,D). Additionally, by validating its expression in TCGA-retrieved ccRCC patients, we found that *miR-134-5p* was dramatically and broadly upregulated in tumor samples (Figure 2E). Kaplan–Meier (KM) survival analyses further suggested that the upregulation of *miR-134-5p* was closely associated with poor survival outcomes in ccRCC patients (Figure 2F), suggesting its oncogenic roles in ccRCC.

### 3.3. miR-134-5p Promotes ccRCC Cell Migration

**Figure 3:**
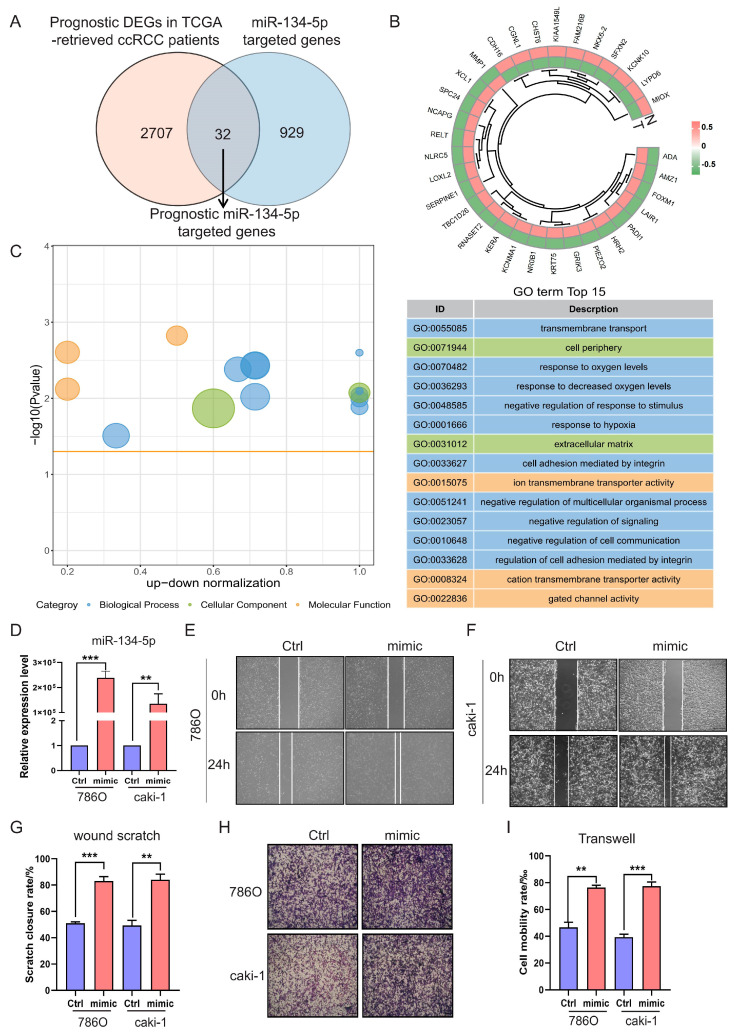
*miR-134-5p* promotes cell migration. (**A**) Venn diagram shows the prognostic target genes of *miR-134-5p*. (**B**) Heatmap shows the expression of the prognostic *miR-134-5p*-argeted genes in TCGA-retrieved normal and tumor specimens. (**C**) Enrichment analysis shows the functional roles of the indicated *miR-134-5p*-argeted genes. (**D**) Overexpression efficiency of *miR-134-5p* mimic in 786O and Caki-1 cells (n = 3). (**E**,**F**) Representative images of wound scratch assay in *miR-134-5p* overexpression 786O cells (**E**) or Caki-1 cells (**F**) in the presence of control or *miR-134-5p* mimic. (**G**) Statistical analysis according to (**E**,**F**) (n = 3). (**H**) Representative images of Transwell assay in *miR-134-5p* overexpression 786O cells (upper panel) or Caki-1 cells (lower panel) in the presence of control or *miR-134-5p* mimic. (**I**) Statistical analysis according to (**H**). Data are shown as means ± SEM. *** *p* < 0.001, and ** *p* < 0.01.

Primarily, miRNAs exert their functions post-transcriptionally by targeting and regulating the expression of their bounded mRNAs [21]. This mechanism is also applicable to mitochondrial microRNAs [22,23]. Therefore, to elucidate the tumorigenic functions of *miR-134-5p*, we utilized multiple databases, including RNAhybrid, miRanda, and TargetScan, to predict the targets of *miR-134-5p*. Consequently, we identified 961 *miR-134-5p*-targeted genes, which are mainly enriched in mitochondrial metabolism and transmembrane transport-related terms (Figure S4A,B and Table S6). To further narrow down and find out prognostic *miR-134-5p*-targeted genes in ccRCC, we also performed differential expression and survival analyses on transcriptome data from TCGA-retrieved ccRCC patients. This analysis revealed 2739 prognostic DEGs in ccRCC (Figure S5A,B and Tables S7 and S8). By intersecting the aforementioned two cohorts, we finally identified 32 prognostic *miR-134-5p*-targeted genes (Figure 3A,B and Table S9).

Next, to investigate the role of these prognostic miR-134-5p-targeted genes, we conducted functional enrichment analysis. Our analysis revealed multiple biological processes, including “transmembrane transport”, “response to hypoxia”, and “cell adhesion” (Figure 3C and Table S10). The gene ontology results were again enriched in the biological processes of metabolism and transmembrane transport-related terms, suggesting that *miR-134-5p* might play an oncogenic role by regulating these functions and ultimately affecting cell adhesion-related functions. To verify whether *miR-134-5p* regulates ccRCC cell migration, we overexpressed *miR-134-5p* mimics. The expression of *miR-134-5p* was significantly increased compared to the control group in both 786O and Caki-1 cell lines (Figure 3D). Subsequently, both wound scratch and Transwell assays demonstrated that the ratio of cell mobility with *miR-134-5p* overexpression was remarkably higher than that of the control in both 786O and Caki-1 cell lines (Figure 3E–I). These results suggest that *miR-134-5p* can enhance ccRCC cell migration capability.

### 3.4. miR-134-5p Plays an Oncogenic Role Through CHST6, SFXN2, and GRIK3

**Figure 4:**
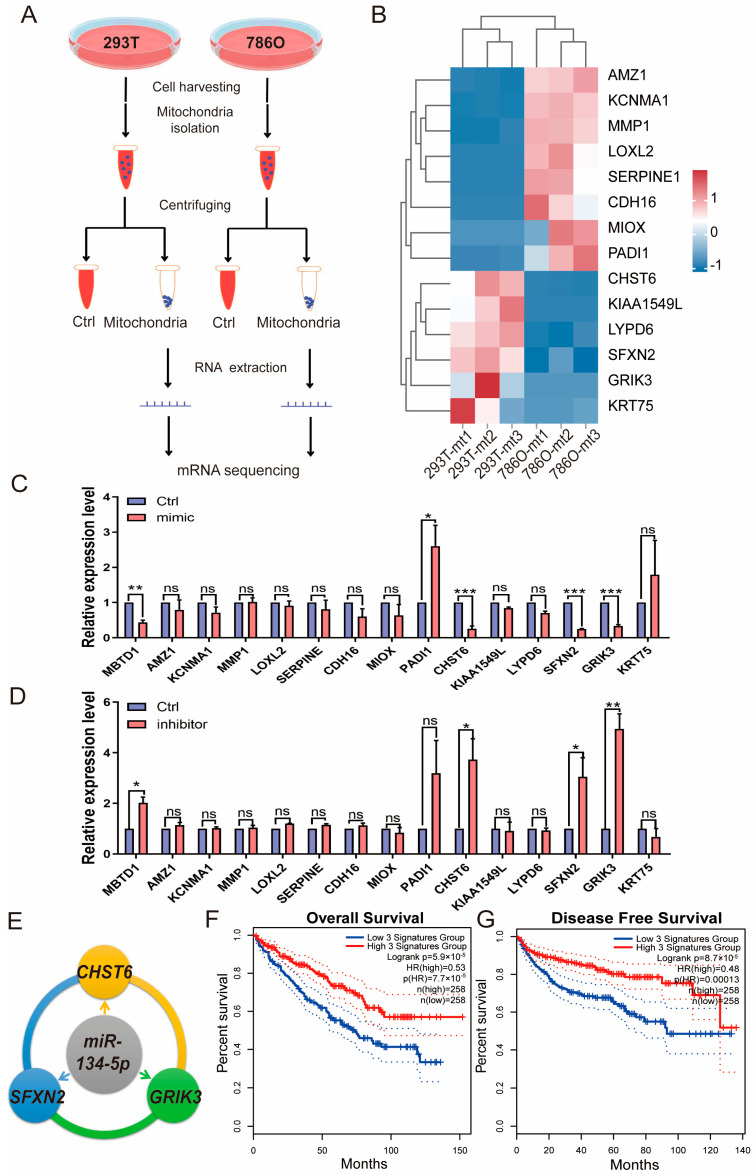
*miR-134-5p* inhibits the mRNA expression of *CHST6*, *SFXN2*, and *GRIK3*. (**A**) A schematic view of experimental outline for identifying the mitochondrial mRNAs in the indicated 293T and 786O cells. (**B**) Heatmap shows the expression of mitochondrial mRNAs in the indicated 293T and 786O cells (n = 3). (**C**) Relative expression of the indicated mitochondrial mRNAs in the present of control or *miR-134-5p* mimic 786O cells (n = 3). (**D**) Relative expression of the indicated mitochondrial mRNAs in the present of control or *miR-134-5p* inhibitor 786O cells (n = 3). (**E**) A schematic view of *miR-134-5p* regulatory networks. (**F**,**G**) Overall (**F**) or Disease free (**G**) survival analysis of *miR-134-5p*-regulated gene signature, including *CHST6*, *SFXN2*, and *GRIK3*, in the TCGA-retrieved ccRCC patients (n (low) = 258, n (high) = 258, HR(OS) = 0.53, HR(DFS) = 0.48). Data are shown as means ± SEM. *** *p* < 0.001, ** *p* < 0.01, * *p* < 0.05, and ns > 0.05.

To identify putative direct targets of *miR-134-5p*, we performed RNA-seq experiments after isolating mitochondria from 786O and 293T cells, as depicted in Figure 4A. Using this approach, we further mined the prognostic DEGs from TCGA-retrieved patient analyses and identified mRNAs that are also expressed in mitochondria (Table S11). Given that *miR-134-5p* is upregulated in 786O cells compared to 293T cells, our focus was on the downregulated genes in 786O cells from the RNA-seq experiments. Our results indicated that *CHST6*, *KIAA1549L*, *LYPD6*, *SFXN2*, *GRIK3*, and *KRT75* might be regulated by *miR-134-5p* (Figure 4B and Table S12). To further validate these findings, we overexpressed a *miR-134-5p* mimic in 786O cells and observed a dramatic decrease in the expression levels of *CHST6*, *SFXN2*, and *GRIK3* (Figure 4C). Conversely, transfection of a *miR-134-5p* inhibitor significantly increased the expression levels of *CHST6*, *SFXN2*, and *GRIK3,* suggesting that their expression could be regulated by miR-134-5p (Figure 4D). Additionally, to ensure the effectiveness of the miR-134-5p mimic and inhibitor, we included *MBTD1*, a literature-reported target of miR-134-5p [24], as a positive control. We found that the *miR-134-5p* mimic significantly decreased *MBTD1* expression, while the *miR-134-5p* inhibitor significantly increased it, further confirming the regulatory roles of *miR-134-5p* in *CHST6*, *SFXN2*, and *GRIK3* (Figure 4C,D). To explore whether the expression signatures of *CHST6*, *SFXN2*, and *GRIK3* are correlated with ccRCC, we conducted KM-plot analysis based on the combined expression signatures of indicated *miR-134-5p* targets. Our results demonstrated that the downregulation of *CHST6*, *SFXN2*, and *GRIK3* was closely associated with poor survival outcomes in ccRCC patients (Figure 4E,F), which aligns well with the oncogenic role of *miR-134-5p* in ccRCC. Overall, our results showed that *miR-134-5p* plays an oncogenic role through regulating the expression of *CHST6*, *SFXN2*, and *GRIK3*.

## 4. Discussion

In this study, we have demonstrated the significant role of mitochondrial microRNA *miR-134-5p* in clear cell renal cell carcinoma (ccRCC), a cancer subtype characterized by its metabolic dysregulation and poor prognosis. Our findings reveal that *miR-134-5p* is predominantly expressed in the mitochondria of ccRCC cells, particularly in the 786O cell line, and that its upregulation correlates with poor survival outcomes in ccRCC patients. This highlights the potential of *miR-134-5p* as a novel biomarker for ccRCC diagnosis and prognosis.

The identification of *miR-134-5p* as a key player in ccRCC progression underscores the importance of mitochondrial miRNAs in cancer biology. Mitochondrial miRNAs, such as *miR-1*, *miR-181c*, and *miR-495*, have previously been implicated in regulating various metabolic pathways and cellular processes [11,12,13]. Our study extends this knowledge by revealing that *miR-134-5p* not only influences mitochondrial functions but also affects cellular migration, a critical aspect of cancer metastasis. The ability of *miR-134-5p* to promote migration in ccRCC cells suggests that it may play a role in enhancing the invasive characteristics of tumor cells, contributing to the aggressive nature of this malignancy.

Furthermore, our mechanistic investigations identified a regulatory network involving CHST6, SFXN2, and GRIK3, the mRNA of which are also predominantly localized in the mitochondria of ccRCC cells. Among them, CHST6 encodes a carbohydrate sulfotransferase that is involved in the modification of glycoproteins and glycolipids [25]. CHST6 has been shown to play a role in cell adhesion and migration, which are crucial processes in cancer metastasis [26,27,28]. SFXN2 is a mitochondrial protein that is implicated in iron homeostasis and mitochondrial function. Research has indicated that SFXN2 is involved in the regulation of mitochondrial respiration and energy production [29,30]. Dysregulation of SFXN2 can lead to altered mitochondrial metabolism, which is often associated with enhanced tumor progression and metastasis [29]. GRIK3 encodes a subunit of the glutamate receptor, which is involved in mediating excitatory neurotransmission. Recent studies have suggested that GRIK3 may also play a role in cancer cell migration and invasion [31,32]. The connection of these genes to both mitochondrial function and cancer migration underscores their relevance in the context of ccRCC.

Mechanistically, miR-134-5p appears to exert its effects by targeting and downregulating the mRNA levels of CHST6, SFXN2, and GRIK3. Interestingly, although CHST6 and GRIK3 proteins are not mitochondrial, our RNA-seq data from mitochondrial fractionation revealed that their mRNAs, along with SFXN2 mRNA, are significantly enriched in the mitochondrial fraction of 786O cells. This finding suggests that the interaction between miR-134-5p and its target mRNAs may occur within the mitochondria, highlighting a potential non-canonical role for mitochondria in mRNA regulation that warrants further investigation. Furthermore, the downregulation of *CHST6*, *SFXN2*, and *GRIK3* upon *miR-134-5p* overexpression indicates that *miR-134-5p* may modulate mitochondrial dynamics and metabolic pathways essential for ccRCC progression.

The identification of *miR-134-5p* as a key player in ccRCC progression also opens up potential therapeutic avenues. Given its role in enhancing the invasive characteristics of tumor cells, targeting *miR-134-5p* could represent a novel therapeutic strategy to inhibit cancer metastasis. By downregulating *miR-134-5p*, it may be possible to restore normal mitochondrial function and metabolic pathways, thereby impeding tumor progression. Furthermore, the development of *miR-134-5p* inhibitors could provide a targeted therapeutic approach to ccRCC, potentially improving patient outcomes. However, further studies are needed to evaluate the efficacy and safety of targeting *miR-134-5p* in therapeutic settings.

Despite the promising implications of our findings, several questions remain to be addressed. First, our study lacks ex vivo validation of the observed effects of miR-134-5p and its targets in patient-derived ccRCC tissues or animal models. Future studies should include ex vivo and in vivo experiments to confirm the functional relevance of miR-134-5p in ccRCC progression and metastasis. Second, the precise mechanisms by which miR-134-5p modulates the expression of CHST6, SFXN2, and GRIK3 need further investigation. For example, it remains unclear whether miR-134-5p directly binds to the mRNA of these targets or exerts its effects through intermediate regulators. Finally, additional studies are needed to evaluate the broader applicability of miR-134-5p as a therapeutic target and its role in mitochondrial miRNA networks across other cancer types.

Nevertheless, our study identifies *miR-134-5p* as a novel mitochondrial miRNA in ccRCC, revealing its potential as a biomarker and therapeutic target. The findings contribute to a growing body of evidence that highlights the significance of mitochondrial miRNAs in cancer biology.

## 5. Conclusions

In conclusion, our study identifies *miR-134-5p* as a novel mitochondrial microRNA that plays a critical role in the progression of ccRCC. By elucidating the regulatory network involving *miR-134-5p* and its target genes *CHST6*, *SFXN2*, and *GRIK3*, we provide valuable insights into the metabolic and migratory dynamics of ccRCC cells. These findings not only highlight the importance of mitochondrial miRNAs in cancer biology but also suggest that *miR-134-5p* could serve as a promising biomarker and therapeutic target in ccRCC.

## Figures and Tables

**Table 1:** Primer sequences for real-time PCR used in this study.

Gene	Forward Primer	Reverse Primer
*ACTB*	CATGTACGTTGCTATCCAGGC	CTCCTTAATGTCACGCACGAT
*GAPDH*	GGGAAACTGTGGCGTGAT	GAGTGGGTGTCGCTGTTGA
*U6*	AAAGCAAATCATCGGACGACC	GTACAACACATTGTTTCCTCGGA
*lncND6*	ATAGGGCAAGGACGCCTCCTAG	GGTAAACTTTAATAGTGTAGGAAGC
*lncCYTB*	ATAGGGCAAGGACGCCTCCTAG	CCAGACAATTATACCCTAGCCA
*AMZ1*	TCAAGGAGCATGAACGGTGG	AGGGAGGAGAACTTGTCCCC
*CDH16*	GTCCCTAGAGCCTATCCACCT	TGCATTCACTTCAAAGGGTCC
*CHST6*	GTTTGATGCCTATCTGCCTTGG	ACGATGCGTAGGTTGAGCG
*GRIK3*	ACACCTTCTACGTGAACCTCT	ACTGTCGTCATAGACCACGGT
*KCNMA1*	TCTTTGCTCTCAGCATCGGTG	CCGCAAGCCGAAGTAGAGAAG
*KIAA1549L*	CCCAAGAAATGGACAGCGGA	GACAGTGCAGGTGGACTTGA
*KRT75*	TTGTAGCCCTGAAAAAGGACG	CAGCTCTGCATCAAAGACTGAG
*LOXL2*	GGGTGGAGGTGTACTATGATGG	CTTGCCGTAGGAGGAGCTG
*LYPD6*	AGTCACCAAACGCTGTGTCC	GTGGGTGCCCATTTGTCTG
*MIOX*	ATCCTCGATACAGCACAGAGC	AGTGGAACCGGATCATGTAGA
*MMP17*	CACTCATGTACTACGCCCTCA	TGGAGAAGTCGATCTGGATGTC
*PADI1*	TGCAGACATGGTCGTATCTGT	GCCCAGAGCTTGGTCTTCC
*SERPINE1*	ACCGCAACGTGGTTTTCTCA	TTGAATCCCATAGCTGCTTGAAT
*SFXN2*	CCATAGGCATCACCCAAGTAGT	CGTGCAGGACCTTGACTTTC

## Data Availability

Datasets in this work are from TCGA database (https://portal.gdc.cancer.gov/, accessed on 15 May 2023). All data generated during this study are included in the manuscript and Supplementary Materials.

## Supplementary Materials

The following supporting information can be downloaded at: https://www.mdpi.com/article/10.3390/biom15030445/s1, **Additional File1: Figure S1.** subcellular distributions of the indicated proteins or RNAs in 293T cells: (A) the subcellular distributions of protein CS, tubulin, and fibrillarin determined by fractionationing assay in 293T cell (n = 3); (B) the subcellular distributions of RNA lncND6, lncCYTB, GAPDH, and NEAT1 determined by fractionationing assay in 293T cell (n = 3); **Figure S2.** characteristics of the indicated sRNA sequencing samples: (A) percentage of each sRNA types in the indicated samples; (C) Pearson correlations between the indicated samples; **Figure S3.** identification of prognostic dysregulated mitomiR in ccRCC: (A) a schematic view of experimental outline for screening pdmt-miRNAs; (B) volcano plot of mitomiR expression profiles from sRNA sequencing of the indicated 293T and 786O cells; (C) volcano plot of mitomiR expression profiles from TCGA-retrieved ccRCC patients and corresponding normal controls; (D) multivariate Cox regression analysis to identify the prognostic mt-miRNAs from TCGA-retrieved ccRCC patients. mt-miRNAs in the indicated figures are referred to as mitomiR; **Figure S4.** identification of miR-134-5p-targeted genes: (A) Venn diagram shows the target genes of miR-134-5p predicted by the indicated RNAhybrid, miRanda, and TargetScan database; (B) enrichment analyses of the functional roles of miR-134-5p-targeted genes; **Figure S5.** identification of prognostic DEGs in TCGA-retrieved ccRCC patients: (A) volcano plot shows the DEGs from TCGA-retrieved ccRCC patients; (C) volcano plot shows the prognostic genes from TCGA-retrieved ccRCC patients. **Additional File2: Table S1**. dysregulated mitomiRs in 786O cells compared with 293T cells; **Table S2.** dysregulated miRNAs in ccRCC retrieved from TCGA; **Table S3.** prognostic dysregulated miRNAs in ccRCC retrieved from TCGA; **Table S4.** Pdmt-miRNAs in the indicated 293T and 786O cells; **Table S5.** the distribution of mitomiRs in the indicated 786O control and mitochondrial component; **Table S6.** miR-134-5p-targeted genes predicted by RNAhybrid, miRanda, and TargetScan; **Table S7.** dysregulated genes in ccRCC retrieved from TCGA; **Table S8.** prognostic DEGs in ccRCC retrieved from TCGA; **Table S9.** prognostic miR-134-5p-target genes; **Table S10.** top 15 GO Terms of the prognostic miR-134-5p-targeted genes; **Table S11.** dysregulated mitochondrial mRNAs in 786O mitochondria compared with other components; **Table S12.** dysregulated mitochondrial mRNAs in 786O cells compared with 293T cells. **Additional File3: File S1:** Original Western blot images.

## Abbreviations

The following abbreviations are used in this manuscript:

mitomiRs	Mitochondrial miRNAs
ccRCC	Clear cell renal cell carcinoma
DMEM	Dulbecco’s modified Eagle’s medium
FBS	Fetal bovine serum
DEGs	Differentially expressed genes
GO	Gene Ontology
qPCR	Quantitative PCR
CS	Citrate synthase
pdmt-miRNAs	Prognostic dysregulated mitomiRs
