# Supplementary material for: Mitochondrial miRNA miR-134-5p Play Oncogenic Role in Clear Cell Renal Cell Carcinoma
Source: Biomolecules. 2025 Mar 20;15(3):445. doi: 10.3390/biom15030445 (PMC11939903; doi:10.3390/biom15030445)
Supplement: Supplementary file 1 [file biomolecules-15-00445-s001.zip › Additional File1_Supplementary Figures-R1.pdf]

Supplementary Figures

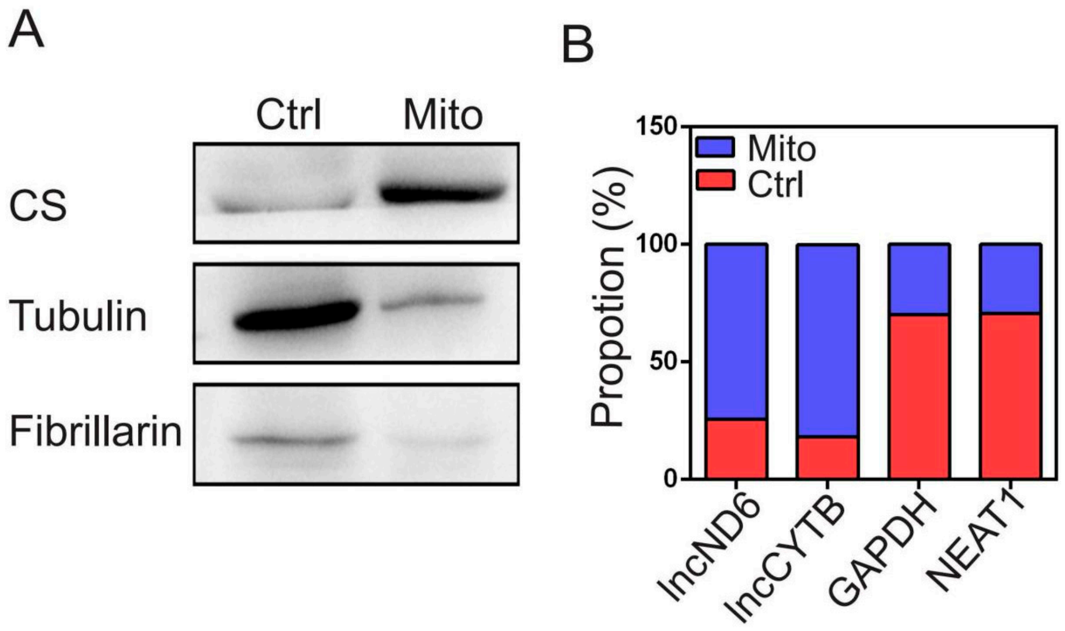

Figure S1. Subcellular distributions of the indicated proteins or RNAs in 293T cells. (A) The subcellular distributions of protein CS, Tubulin, and Fibrillarin determined by fractionationing assay in 293T cell (n=3). (B) The subcellular distributions of RNA *lncND6*, *lncCYTB*, *GAPDH*, and *NEAT1* determined by fractionationing assay in 293T cell (n=3).

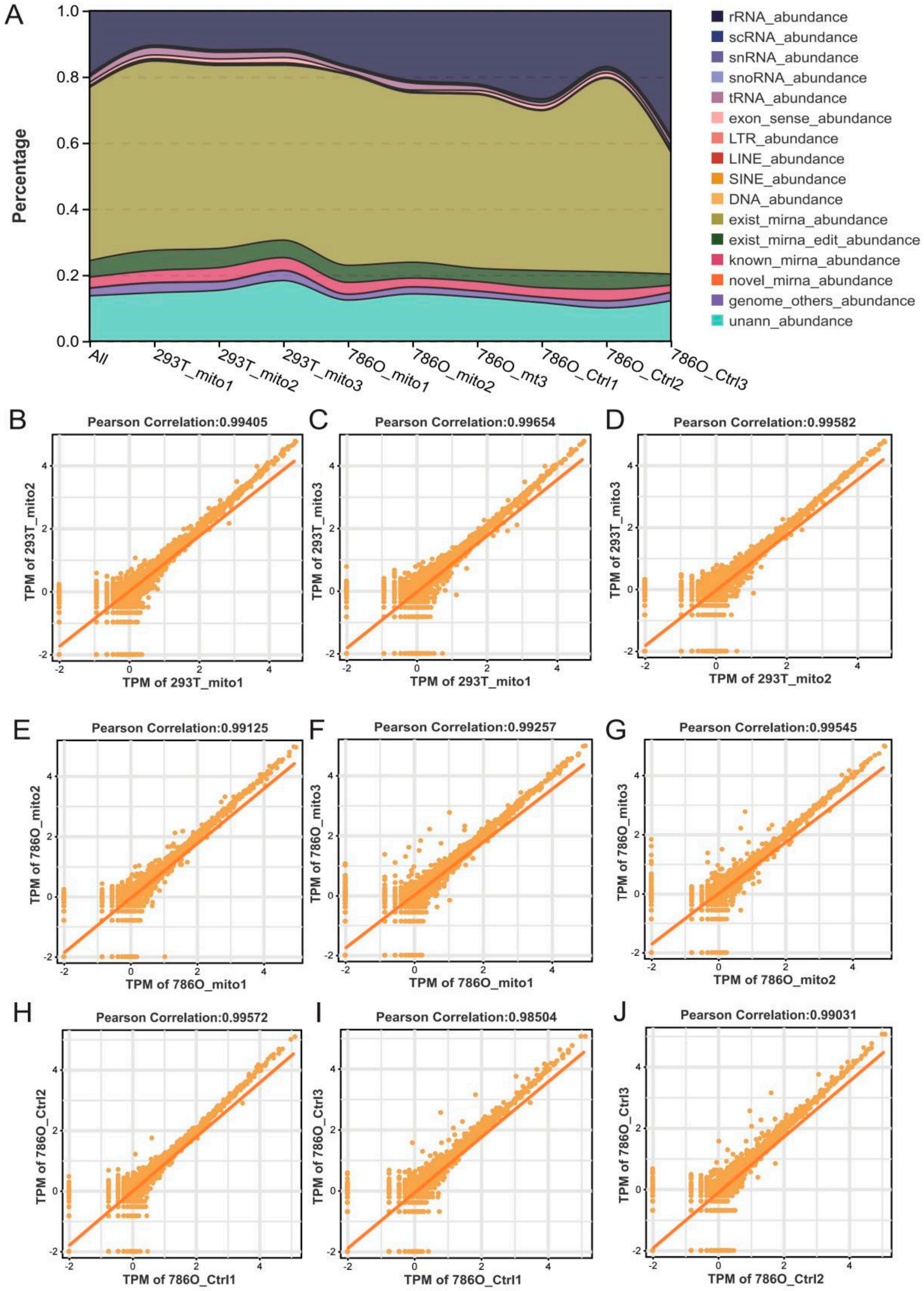

Figure S2. Characteristics of the indicated sRNA sequencing samples. (A) Percentage of each sRNA types in the indicated samples. (B-J) Pearson correlations between the indicated samples.

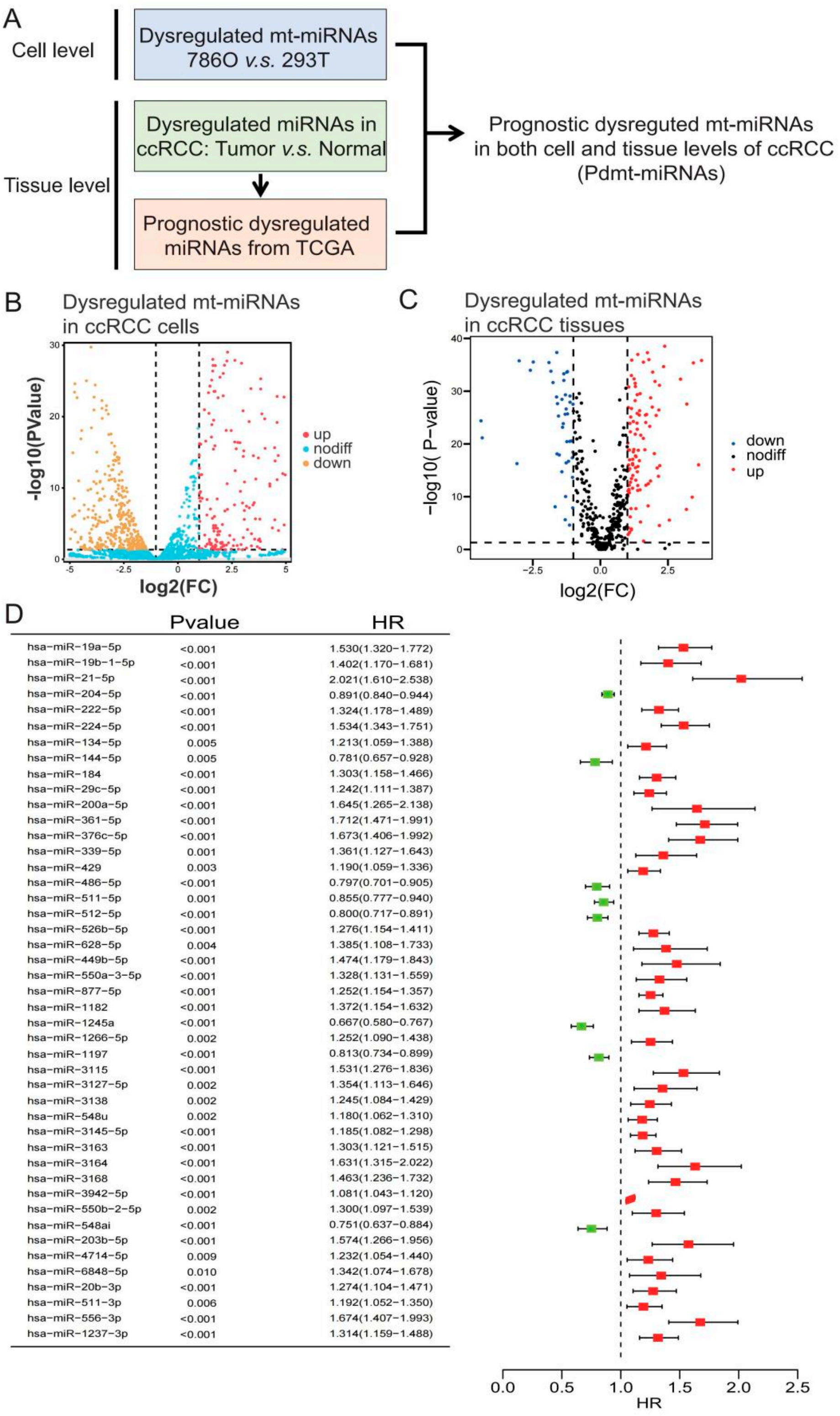

Figure S3. Identification of prognostic dysregulated mitomiR in ccRCC. (A) A schematic view of experimental outline for screening pdmt-miRNAs. (B) Volcano plot of mitomiR expression profiles from sRNA sequencing of the indicated 293T and 786O cells. (C) Volcano plot of mitomiR expression profiles from TCGA retrieved ccRCC patients and corresponding normal controls. (D) Multivariate cox regression analysis to identify the prognostic mt-miRNAs from TCGA retrieved ccRCC patients. mt-miRNAs in the indicated figures refered as mitomiR.

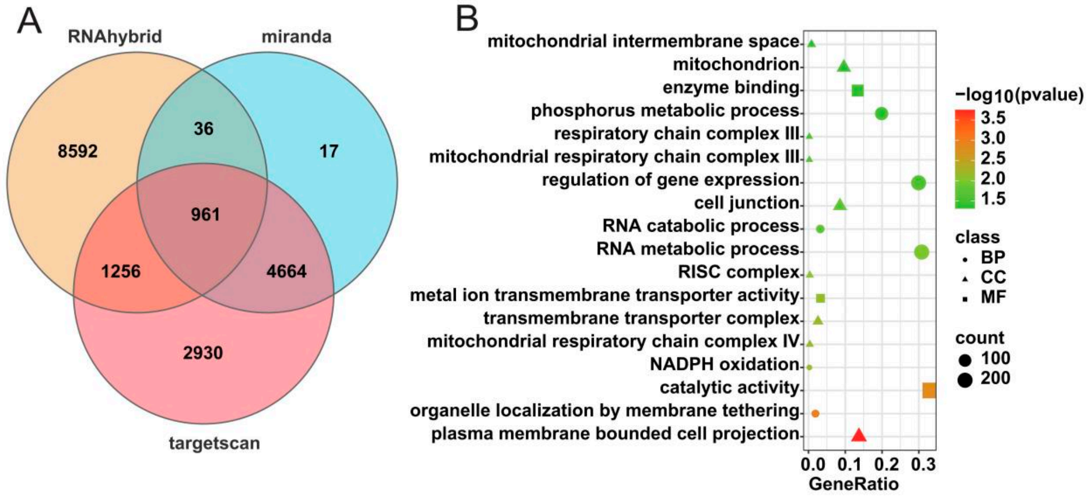

Figure S4. Identification of *miR-134-5p* targeted genes. (A) Venn diagram shows the target genes of *miR-134-5p* predicted by the indicated RNAhybrid, miranda, and targetscan database. (B) Enrichment analyses of the functional roles of *miR-134-5p* targeted genes.

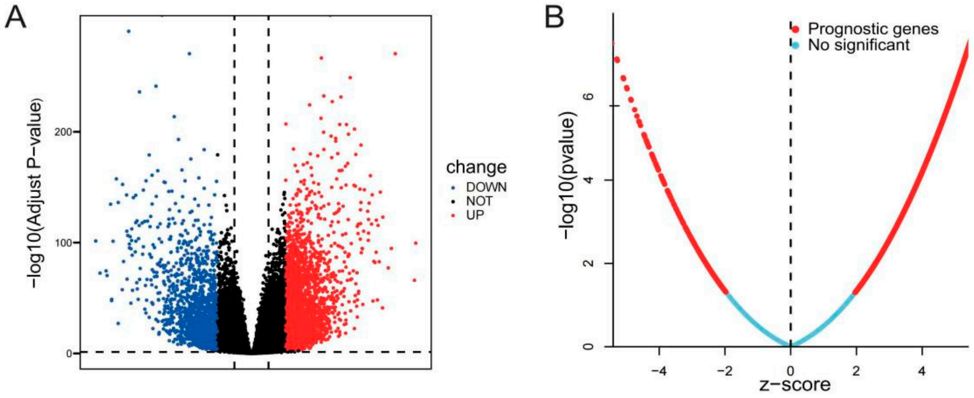

Figure S5. Identification of prognostic DEGs in TCGA-retrieved ccRCC patients. (A) Volcano plot shows the DEGs from TCGA-retrieved ccRCC patients. (C) Volcano plot shows the prognostic genes from TCGA-retrieved ccRCC patients.
